# Effect of Ceramic and Dentin Thicknesses and Type of Resin-Based Luting Agents on Intrapulpal Temperature Changes during Luting of Ceramic Inlays

**DOI:** 10.3390/ijms24065466

**Published:** 2023-03-13

**Authors:** Dóra Kincses, Dóra Jordáki, Donát Szebeni, Sándor Kunsági-Máté, József Szalma, Edina Lempel

**Affiliations:** 1 Department of Restorative Dentistry and Periodontology, Faculty of Dentistry, University of Pécs Medical School, PTüzér Street 1, 7623 Pécs, Hungary; 2 Department of Organic and Medicinal Chemistry, Faculty of Pharmacy, University of Pécs, Honvéd Street 1, 7624 Pécs, Hungary; 3 János Szentágothai Research Center, Ifjúság Street 20, 7624 Pécs, Hungary; 4 Department of Oral and Maxillofacial Surgery, Faculty of Dentistry, University of Pécs Medical School, Tüzér Street 1, 7623 Pécs, Hungary

**Keywords:** pulpal temperature, indirect restoration, dentin thickness, ceramic thickness, preheated resin composite, adhesive cement

## Abstract

The adhesive cementation of ceramic inlays may increase pulpal temperature (PT) and induce pulpal damage due to heat generated by the curing unit and the exothermic reaction of the luting agent (LA). The aim was to measure the PT rise during ceramic inlay cementation by testing different combinations of dentin and ceramic thicknesses and LAs. The PT changes were detected using a thermocouple sensor positioned in the pulp chamber of a mandibular molar. Gradual occlusal reduction obtained dentin thicknesses of 2.5, 2.0, 1.5, and 1.0 mm. Light-cured (LC) and dual-cured (DC) adhesive cements and preheated restorative resin-based composite (RBC) were applied to luting of 2.0, 2.5, 3.0, and 3.5 mm lithium disilicate ceramic blocks. Differential scanning calorimetry was used to compare the thermal conductivity of dentin and ceramic slices. Although ceramic reduced heat delivered by the curing unit, the exothermic reaction of the LAs significantly increased it in each investigated combination (5.4–7.9 °C). Temperature changes were predominantly influenced by dentin thickness followed by LA and ceramic thickness. Thermal conductivity of dentin was 24% lower than that of ceramic, and its thermal capacity was 86% higher. Regardless of the ceramic thickness, adhesive inlay cementation can significantly increase the PT, especially when the remaining dentin thickness is <2 mm.

## 1. Introduction

Posterior indirect partial restorations (inlay, onlay, overlay) are widely used in dental clinical practice to overcome issues resulting from the use of direct resin-based composites (RBCs) [1,2,3].

The adhesive cementation of ceramic inlays is recommended to improve the esthetic and mechanical properties of the restoration [4,5,6,7,8]. Resin-based adhesive cements can be classified as self-cured, dual-cured, or light-cured based on their polymerization mechanism [9]. An alternative innovation is the use of chairside preheated conventional restorative RBCs as a luting agent for indirect ceramic restorations. Owing to their reduced viscosity, low film thickness and good adaptation can be achieved [10]. In addition to color stability, favorable mechanical and physical properties are further benefits as a result of their high filler load [11,12,13]. An increased pre-polymerization temperature can improve the monomer-to-polymer conversion; however, this might be compromised during the luting procedure because of the rapid cooling of the luting RBC before it is light-cured [14,15,16].

The durability of adhesively bonded restorations depends on the degree of conversion of the adhesive cement [17,18]. However, indirect ceramic restorations attenuate the light passing through them [19]. The interposed material might impair the mechanical and esthetic properties of the luting agent caused by the reduced degree of conversion, thus compromising the durability of the indirect restoration [20,21]. To overcome or attempt to compensate for this drawback, the light intensity should be sufficiently high or the exposure time should be as long as possible, considering light attenuation as a function of the restoration thickness [20,22]. However, an increase in irradiance delivered from the curing unit or upon extending the exposure time as a strategy to enhance the polymerization degree might cause an unfavorable temperature rise within the pulp chamber [23]. The temperature rise during polymerization is due to the absorption of energy by the irradiated objects together with the heating of the curing unit itself [24]. However, the polymerization of resin-based materials is an exothermic reaction that leads to further heat generation [25,26]. Bouillaguet et al. [24] reported that, by using infrared imaging, the highest temperature increases were recorded inside the RBC material and not outside the tooth during photocuring. The exothermic reaction is a material-, consistency-, and thickness-dependent phenomenon [26]. Although the majority of the heat generated during RBC polymerization is dissipated, the increase in pulpal temperature may exceed the putative pulpal damage threshold [27]. Several studies have consistently reported that the remaining dentin thickness is a critical factor in relation to the intrapulpal temperature increase due to the heat dissipating effect [23,28,29]. According to the second law of thermodynamics, during light curing, heat flows from the external tooth surface or polymerizing material to the pulp chamber as the temperature difference is equalized by diffusion [30]. The thermal conductivity of human dentin was calculated at approximately 0.36–0.67 W/mK by de Magalhães et al. [31]. Although heat transfer or heat flux occurs at a lower rate in dentin, inducing a thermal insulating effect, the potential for pulpal damage is expected to be great in deep cavities where the tubular surface area increases and the light attenuation effect is weak [32,33,34]. Thus, clinically, it would be optimal for dentin preservation, or continuous high-energy output photo curing should be avoided to protect pulp tissues from thermal injury [35]. Onisor et al. [36] conducted active cooling to reduce the heat during prolonged polymerization employed for luting indirect adhesive restorations with light-cured materials. Additionally, the shielding effect of dentin resulted in a lower pulpal temperature increase than that of the interposed ceramic restoration during light curing [35]. In addition to the interposed materials and the distance between the cavity floor and the pulp, the effect of pulpal blood circulation, volume, and perfusion of the fluid in the dentinal tubules as well as in the surrounding tissues play important roles in heat conduction and protection against the rise in pulpal temperature [37,38]. The putative pulpal damage threshold was based on the study conducted by Zach and Cohen. A temperature rise in pulpal tissues of 5.5 °C may lead to irreversible changes [39,40]. In support of the above observation, a recent in vivo study showed that increased pulpal temperatures may induce inflammatory reactions, even if the temperature rise does not exceed the previously defined 5.5 °C threshold [41].

Although several investigations have been conducted on the effects of the light curing unit, resin composite type, and remaining dentin thickness on the pulpal temperature rise, data are lacking in the dental literature regarding the effects of different dentin thicknesses on pulpal temperature change during cementation of different thicknesses of indirect ceramic restorations with adhesive resin cements or preheated restorative RBCs [25,26,33,42,43,44,45]. Furthermore, reliable, comparative data on the thermophysical properties of dentin and ceramic are essential to obtain precise calculations of the thermal changes in teeth and provide safer dental procedures, such as ceramic inlay cementation.

Therefore, this study compared, in vitro, the intrapulpal thermal changes resulting from cementation of ceramic inlay with light- and dual-curing adhesive resin cements and preheated sculptable submicron restorative RBCs. The aim of this study was to assess the influence of simultaneously variable dentin and ceramic layer thicknesses on pulpal temperature rise, supplemented by a qualitative comparison of the thermal properties between dentin and ceramics.

The null hypotheses of the study were threefold: (1) there is no difference in pulpal temperature change using different luting agents during ceramic inlay cementation; (2) there is no significant influence of ceramic and dentin layer thicknesses on pulpal temperature rise; and (3) there is no significant difference between the thermal conductivity and heat capacity of dentin and ceramics.

## 2. Results

**Figure 1:**
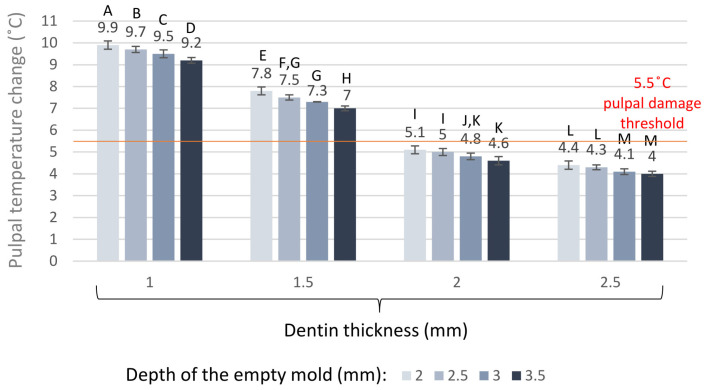
Intrapulpal temperature changes during light exposure of dentin adhesive through the different dentin thicknesses using the empty molds. Different capital letters (A–M) indicate statistically significant differences according to the one-way ANOVA and Tukey’s post-hoc test.

**Figure 2:**
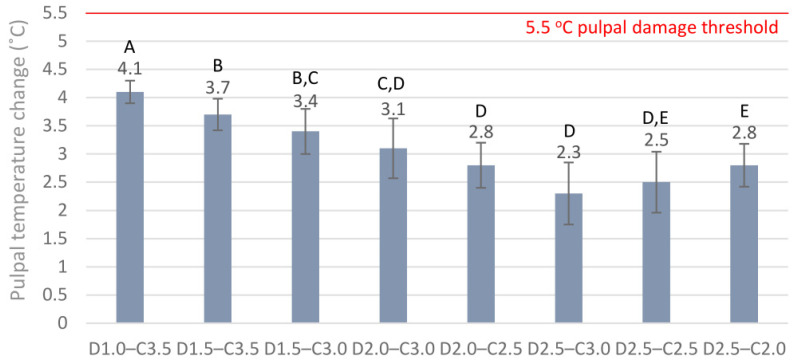
Pulpal temperature changes during light exposure through eight different combinations of dentin (D1.0, 1 mm; D1.5, 1.5 mm; D2.0, 2.0 mm; D2.5, 2.5 mm) and ceramic thicknesses (C2.0, 2.0 mm; C2.5, 2.5 mm; C3.0, 3.0 mm; C3.5, 3.5 mm) without luting cement. Different capital letters (A–E) indicate statistically significant differences according to the one-way ANOVA and Tukey’s post-hoc test.

The maximum radiant exitance of the LED LCU was 1550 ± 15 mW/cm^2^. The delivered maximum incident radiant exposure with a 40 s exposure duration was 62 ± 0.6 J/cm^2^. The radiant exitance was reduced by 20% (1240 ± 12 mW/cm^2^) by the 6 × 6 mm orifice; thus, the radiant exposure with 40 s exposure duration was 49.6 ± 0.6 J/cm^2^, which was delivered to the top of the ceramic specimens. The 2.0 mm, 2.5 mm, 3.0 mm, and 3.5 mm distances between the light guide tip and the radiometer sensor and the limited orifice of the mold significantly decreased the radiant exposure. Through the empty 2.0 mm, 2.5 mm, 3.0 mm, and 3.5 mm deep molds, the radiant exposures decreased by 42% (36 ± 0.4 J/cm^2^), 45% (34 J/cm^2^ ± 0.3), 48% (32.2 ± 0.3 J/cm^2^), and 51% (30.4 ± 0.3 J/cm^2^), respectively. The 2.0 mm, 2.5 mm, 3.0 mm, and 3.5 mm thick ceramics further decreased the radiant exposures by 67% (20.5 ± 0.4 J/cm^2^), 70% (18.6 J/cm^2^ ± 0.3), 73.2% (16.6 ± 0.3 J/cm^2^), and 76% (14.9 ± 0.3 J/cm^2^), respectively. The intrapulpal thermal changes induced by the 40 s light exposure of dentin adhesive through the 1.0 mm, 1.5 mm, 2.0 mm, and 2.5 mm dentin thicknesses, using the 2.0 mm, 2.5 mm, 3.0 mm, and 3.5 mm deep empty molds, are presented in Figure 1, representing the insulating effect of the dentin without the ceramic blocks and luting agents. The thermal effect of the LCU through the eight combinations of different thicknesses of dentin and ceramic assemblies without luting agents is presented in Figure 2. None of the combinations approached the critical 5.5 °C threshold. Increased dentin thickness showed a more pronounced insulating effect than ceramic thickness.

**Figure 3:**
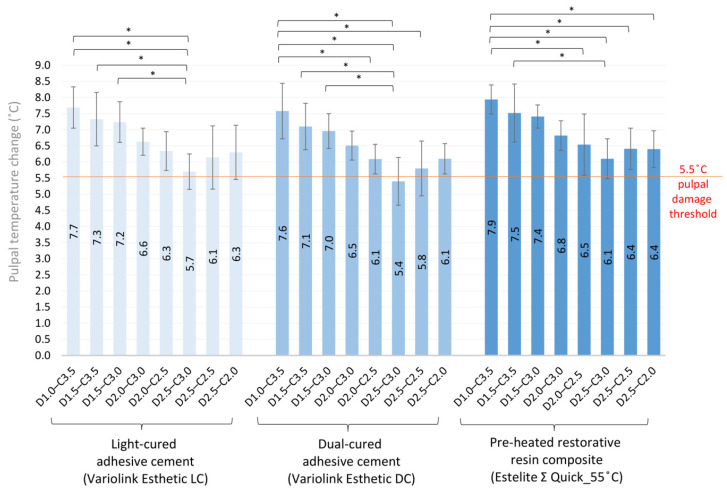
Pulpal temperature changes during cementation of eight different combinations of ceramic and dentin thicknesses cemented with light-cured and dual-cured adhesive cements and with the preheated restorative resin composite (* mark demonstrates statistically significant difference between groups according to the one-way ANOVA and Tukey’s post-hoc test).

**Figure 4:**
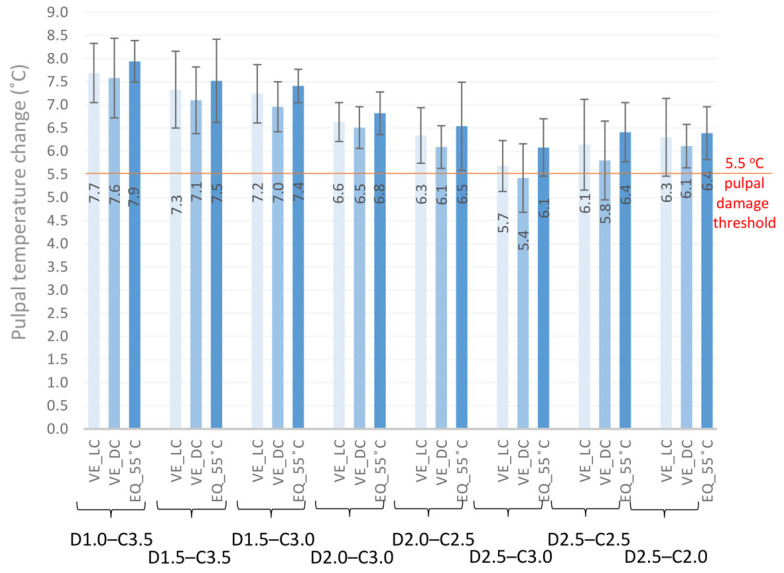
Comparisons of the pulpal temperature changes caused by the polymerization of light-cured and dual-cured adhesive cements and by the preheated restorative resin composite through eight different combinations of dentin and ceramic thicknesses (one-way ANOVA and Tukey’s post-hoc test did not result in statistically significant differences between the tested groups).

Luting the ceramic blocks into cavities of different depths with light-cured and dual-cured adhesive cements, or with the restorative RBC preheated to 55 °C, increased the pulpal temperature significantly (Figure 3). Subtracting the temperature rise caused by the LCU from the thermal change in the pulp chamber induced by the luting agent provides an estimation of the heat generated by the exothermic reaction. According to this calculation, which does not account for the thermal transfer between the thermodynamic system and its environment, the preheated RBC elevated the pulpal temperature to the highest value, although a statistically significant difference was not detected between the luting materials (Figure 4).

**Figure 5:**
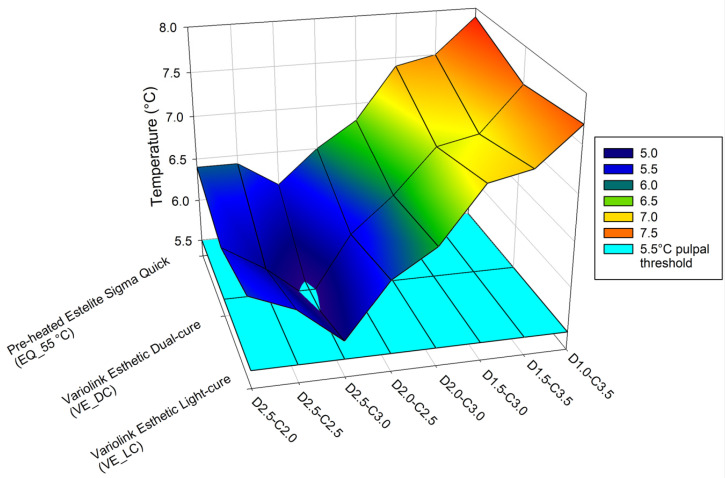
Changes in pulpal temperature during polymerization of the light-cured and dual-cured resin cements and preheated resin composite through different thicknesses of dentin and ceramic blocks representing an inlay. The light blue layer demonstrates the 5.5 °C temperature change thought to be associated with pulpal damage.

The mesh figure shows that the dentin thickness below 1.5 mm is the most critical for the heat insulating effect (Figure 5).

The multivariate general linear model revealed that the dentin thickness had the greatest effect on the pulpal temperature changes (F (3, 96) = 6.02, *p* = 0.001) followed by the effect of luting material (F (2, 96) = 4.29, *p* = 0.02). The effect size was considered to be large for the dentin thickness (partial ƞ^2^ = 0.16) and medium for the effect of material (partial ƞ^2^ = 0.08). The effect of ceramic thickness on the pulpal temperature rise was considered to be insignificant according to the results of the general linear model (F (3, 96) = 2.28, *p* = 0.09), although the eta-squared indicated a medium effect (partial ƞ^2^ was 0.07). However, there was no statistically significant three-way interaction between material, dentin thickness, and ceramic thickness [F (14, 96) = 0.06, *p* = 1.0; partial ƞ^2^ = 0.009]. According to the linear curve-fitting model, the data regarding the dentin and ceramic thicknesses and the tested luting agents allowed us to predict the behavior of the data series. The adjusted R-square statistics revealed higher values for dentin (R^2^ = 0.47) and ceramic thickness (R^2^ = 0.41), indicating a better fit; meanwhile, the R^2^ value for the materials was 1%.

**Figure 6:**
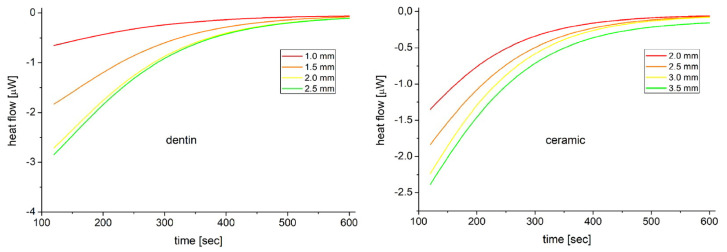
Heat flow associated with the dentin (left) or ceramic (right) samples plotted as a function of the time. Three scans of each sample were averaged then fitted using the exponential saturation function.

The thermal properties of the samples were evaluated by DSC measurements. The heat capacities were measured directly with the Calisto software by calculating the area of the curve, where the heat flow was plotted as a function of time until the curve reached the saturated region. Considering the mass of the samples and the area of the heating curves, the heat capacity of dentin was found to be 86% larger than the heat capacity of ceramic. To compare the heat conductivities, the time constant for the achievement of thermal equilibrium was applied. The average time constants of the dentin and ceramic samples were measured as 165.72 s and 133.33 s, respectively. The pre-exponential factors associated with the samples with increasing thickness and mass are −1.35 µW, −4.08 µW, −6.08 µW, and −6.39 µW, or −7.13 µW, −9.13 µW, −11.38 µW, and −12.02 µW in respect to the dentin or ceramic samples. Calculating the thermal conductivity difference using the ratio of time constants, it was found to be approximately 24% lower in the case of dentin samples compared to ceramic specimens (Figure 6).

## 3. Discussion

In this in vitro study, the influence of the polymerization of light-cured and dual-cured adhesive resin cements and preheated restorative RBCs on the thermal change in the pulp chamber was investigated using different thicknesses of dentin and ceramic blocks imitating inlays. The results showed that, in the case of the investigated ceramic–dentin combinations, the cementing agents increased the intrapulpal temperature above the considered critical 5.5 °C. Furthermore, a qualitative comparison of thermal conductivity and capacity showed differences between the dentin and the ceramic. Therefore, all the tested null hypotheses were rejected. Our findings are consistent with the results of other studies regarding the shielding effect of the interposed ceramic and the remaining dentin thickness on pulpal temperature rise, and the material-dependent temperature-increasing effect of the polymerization of RBCs [25,26,35]. Thus, even though the interposed ceramic inlay and the remaining dentin thickness attenuate the light intensity of the curing unit and the delivered energy during the polymerization process, the exothermic temperature rise associated with the adhesive luting agent may jeopardize pulp health.

To eliminate any effects that may arise from structural differences and thermal properties of the tooth structure, this study was carried out on a representative permanent third molar without the use of acid conditioning of tooth and any ceramic surface treatment for all experimental groups. This provided the same tooth conditions for each measurement. However, the tooth differences regarding thermal changes are not accounted for in this model. Furthermore, this technique has limitations, as heat dissipation by pulpal, periodontal, and osseous circulation is not reproduced [46,47]. Thus, the temperature changes measured in this study cannot be directly applied under in vivo conditions. Despite the absence of blood circulation in vital tissues, this study provides important information regarding the magnitude of temperature change in a model system when a ceramic inlay is cemented in cavities of different depths using different adhesive luting agents.

In this study, a thermocouple was used for the instantaneous observation of temperature changes during ceramic inlay cementation under laboratory conditions. The use of a thermocouple is a simple and well-known method for measuring differences in temperature during dental treatments [25,26]. Compared to more modern methods, such as infrared thermography, thermocouples can measure similar values with differences of less than 1 °C in favor of infrared measurements [24].

It has been reported that dental pulp is vulnerable to temperature changes despite its high vascularization, which is the main regulatory system for heat distribution and which is capable of dissipating external thermal stimuli transferred to the dentin–pulp complex [48]. The clinical relevance of increased intrapulpal temperature is that it is a potential risk factor for thermal pulp damage. According to Zach and Cohen, a 5.5 °C temperature rise is critical and may cause irreversible pulpal damage [39]. In line with the previous experiment, Pohto and Scheinin reported that the critical temperature for reversible pulp damage was between 42 °C and 42.5 °C [49]. Although the pathological threshold of pulpal temperature rise was determined to be approximately 5.5 °C, a more recent study did not find an average increase of 11.2 °C to compromise pulp health significantly [50]. However, a histomorphometric analysis using a clinically valid 3-dimensional organotypic ex vivo model showed an immediate reduction in cell number with a temperature increase of 5.5 °C or greater, which was dependent on exposure time [40]. Additionally, immunohistochemical changes were observed at a temperature increase of 6 °C or higher [40].

Despite attempts to simulate in vivo conditions using several reliable methods, a wide range of intrapulpal temperature increases has been reported in vitro during photocuring [27]. To provide the blue light required for the polymerization of resin-based luting agents, a second-generation LED LCU was used in this study with a radiant exitance of 1550 ± 10 mW/cm^2^ in the wavelength range of 420–480 nm. According to previous studies, the intensity and duration of the applied light were the most crucial factors for the pulpal temperature rise [51,52]. The results of this study confirm the above statement because light curing without the interposition of a ceramic inlay and resin-based luting agent increased the pulpal temperature (ΔT = 4.0–9.9 °C) by a significant degree, depending on the dentin thickness, with an inverse correlation. Although a thin layer of dentin adhesive was used during light curing without ceramic blocks and luting agents, the heat measured was predominantly from the curing unit. A pilot study was undertaken to assess the thermal effect of the dentin adhesive layer, and a negligible, insignificant (0.1–0.2 °C) temperature difference was detected during light curing with or without the adhesive.

Because the monomer-to-polymer conversion of an RBC is a function of the applied total energy during photocuring, it is advisable to increase the delivered radiant exposure for a higher degree of conversion [53]. This is highly relevant to adhesive luting of indirect restorations, where a high radiant exposure is needed for proper curing of the luting resin-based material underneath a certain thickness of ceramic [34]. The reciprocal relationship between the power output and exposure duration provides an opportunity to increase either irradiance or exposure time, resulting in a higher delivered radiant exposure [53]. It was affirmed that extended irradiation has a greater effect on the depth of cure than increasing the light irradiance of the curing unit [54]. While the increased delivered radiant exposure is indispensable to the acceptable polymerization of the adhesive luting agent, a strong positive correlation was found between the radiant exposure and the intrapulpal temperature rise [26,55]. In this study, an extended exposure time (40 s) with a light irradiance of 1550 mW/cm^2^ resulted in 62 J/cm^2^ of delivered radiant exposure. Onisor et al. [36] investigated the effect of extended exposure times on intrapulpal temperature increase through 1 mm of the remaining dentin thickness, interposing a 3 mm ceramic onlay and 0.3 mm of previously polymerized RBC luting agent. They found a maximal temperature difference of 4.3 °C during the 3 × 20 s of extended irradiation, delivering ~60 J/cm^2^ of total energy. Although the study design and the type of curing units are slightly different, these results are in line with our findings with a similar D1–C3.5 combination, where the intrapulpal temperature rise was 4.1 °C without the exothermic reaction of the luting agent and the delivered energy density was 62 J/cm^2^. In addition to energy density, other characteristics of the photocuring unit affect the amount of heat generated within the pulp [36,46,56]. While the pulpal temperature in this study remained below the critical value of 5.5 °C without luting agents, the tested adhesive cements significantly increased it because of their exothermic reaction. Our study design ensured uniform conditions, allowing the comparison of exothermic thermal changes between different resin-based luting agents. The exothermic reaction was proportional to the amount of resin matrix, and it was found that the inorganic fillers have an impact on heat diffusion within the material by their capacity to absorb external and internal energy [26,57,58]. The multivariate general linear model revealed that the material factor had a medium impact on the pulpal temperature rise. A tendency was observed when comparing the effects of the tested luting agents on temperature change, although there was no detectable statistically significant difference among the study groups. The highest temperature was measured with the preheated RBC in all the tested groups, followed by the light-cured adhesive cement, and the lowest temperature increase was observed with the dual-cured luting agent. Although the restorative RBC was preheated to 55 °C, it only increased the pre-polymerization temperature of the pulp by ~3 °C, and further cooling was detected during the cementation procedure before light curing. After removal from the warming device, the preheated RBC was reported to suffer from rapid cooling, which may compromise the degree of conversion [16]. Although the temperature-raising effect was less than expected owing to the rapid cooling and heat absorption by the ceramic and dentin, these results suggest the potential hazard to pulp health owing to their higher thermal effect. Regarding the composition of the investigated light- and dual-cured resin cements, they had the same resin matrix/filler ratio; however, the dual-cured cement showed a slightly, but not significantly, lower temperature rise in each tested group compared to that of the light-cured resin cement. Dual-cured resin cements are supposed to compensate for decreased light transmission and may be more efficient at monomer-to-polymer conversion, even with increased ceramic thicknesses [59,60]. In their systematic review, David-Pérez et al. found that dual-cured resin cements fail to achieve the same degree of conversion as light-cured cements with up to 2 mm of interposed ceramic thickness [61]. Our results are indirectly in line with these findings because the polymerization process is proportional to the exothermic reaction, which results in a slightly lower pulpal temperature rise in the dual-cured resin cement [57].

Thermal transfer to the pulp is strongly dependent on the thickness of the remaining tooth structure [35]. The thermal conductivities of enamel and dentin are ~0.81 W/mK and ~0.48 W/mK, respectively, which are considered low [62]. Low thermal conductivity is equivalent to high insulating capability; thus, the pulp is protected from noxious thermal irritation if the tooth is intact [62]. The advantageous thermal conductivity may be explained by the porous tubular microstructure in the dentin layer, which is a mineralized connective tissue with an organic matrix of collagenous proteins [33]. However, during cavity preparation for direct or indirect restorations, the enamel and DEJ are partially removed, and the dentin thickness is reduced according to the extent of the caries or depending on the special cavity design. Hard tissue removal during cavity preparation and several steps of the adhesive restorative procedure (i.e., polymerization of the adhesive layer and the RBC/adhesive luting agent, and the polishing procedure) may cause thermal damage due to the weakened thermal insulation effect, especially in younger patients with wider dentinal tubules [63,64]. The present study investigated four thicknesses (1.0 mm, 1.5 mm, 2.0 mm, and 2.5 mm) of the remaining dentin layer. For dentin thickness below 2.0 mm, the heat transmitted by the curing unit increased the intrapulpal temperature above the 5.5 °C limit. These results are consistent with previous finding that showed a strong relationship between the thickness of the dentin and the intrapulpal temperature increase [29]. Similar to our findings, Kuo et al. concluded that there is a risk of damaging the pulp when the dentin thickness is less than 2.0 mm and the overall thickness of the dentin–ceramic assembly is less than 3.5 mm [35]. However, according to other studies, the dentin thickness (0.5 vs. 1.0 mm) had an insignificant role in the pulpal temperature increase compared to the curing unit type [28]. In addition to the dentin insulating effect, the already polymerized thin dentin adhesive may serve as a further protective layer during the cementing procedure. However, our pilot study showed that the thermal insulating effect of dentin–ceramic assemblies is not affected by the presence or absence of a polymerized adhesive layer. In the present study, partial eta-squared statistics revealed that, among the investigated factors—such as dentin thickness, ceramic thickness, and the type of luting material—the remaining dentin layer had the most pronounced effect on the pulpal temperature change values, and the effect size was considered to be large. The linear regression model revealed a 47% value for the coefficient of determination and predicted a decreasing effect of thickness on temperature as the dentin thickness exceeded 2 mm. Even though the thinnest dentin was combined with the thickest ceramic, the highest intrapulpal temperature was detected in all measurements, regardless of the use of the luting agents.

In contrast, the effect of ceramic thickness on pulpal temperature rise was considered insignificant according to the results of the general linear model, although the partial eta-squared indicated a medium effect. This result demonstrates that the shielding effect of the ceramic is not as great as that of dentin, although an inverse relationship between the ceramic thickness and temperature rise is evident, which is consistent with the findings of a previous study [35]. The regression curve fit for temperature as a function of different ceramic thicknesses showed a linear energy loss with increasing ceramic thickness, which reflects the light attenuation occurring through an absorptive/scattering medium. The value of the coefficient of determination for ceramic thickness was found to be 41% in our linear regression model. The differences in temperature changes caused by the thermal shielding effect of dentin and ceramic can be explained by their distinct thermal conductivities. It is higher for silica-based ceramics, which is approximately 1.7 W/mK, compared to the thermal conductivity of the dentin (~0.48 W/mK) [65]. Increasing the inlay thickness may result in a proportional removal of tooth hard tissues by decreasing the thickness of the remaining enamel and dentin. Although the shielding effect is proportional to increasing ceramic thickness, a decreasing dentin thickness has a stronger inversely proportional effect on the temperature increase within the pulp chamber. These results are supported by the DSC measurements in this study, which revealed a 24% lower thermal conductivity of the dentin than that of the ceramic. However, the thermal capacity of the dentin was calculated to be 86% more compared to the investigated lithium disilicate ceramic. The importance of this result lies in the ability of dentin to store large amounts of heat and then dissipate it slowly, reducing the sudden thermal effects on the pulp. However, considering the multiple thermal effects during adhesive restorative treatment, the gradually increasing temperature of the dentin may conduct more heat towards the pulp during heat dissipation. According to these findings, it is advisable that more dentin should be preserved during cavity preparation to protect the pulp from undesirable temperature increases. Furthermore, it should provide more time for heat dissipation between treatment steps, which can have a thermal effect on the pulp, to avoid heat accumulation in the dentin.

## 4. Materials and Methods

### 4.1. Resin-Based Luting Agents, Ceramic Blocks, and Radiant Exposure

In this in vitro study, the effects of three resin-based luting agents—Variolink Esthetic LC (VE_LC) light curing, Variolink Esthetic DC (VE_DC) dual-curing adhesive resin cement, and preheated sculptable submicron filled restorative RBC, Estelite Sigma Quick (EQ_55 °C)—on pulpal temperatures were analyzed. The brands, manufacturers, and chemical compositions are listed in Table 1.

Highly translucent A2 shade lithium disilicate ceramic blocks (6 × 6 mm) were fabricated from ceramic ingots (GC Initial LiSi Press; GC Europe, Leuven, Belgium) using the heat-pressed method and were then fired and glazed from one side according to the manufacturer’s instructions. To achieve an even smoother surface, 220-, 400-, and 600-grit water-cooled sandpaper was used to finish the specimens, followed by polishing with a two-step rubber diamond polisher (fine, 8–32 μm grit size, Kenda Nobilis, Kenda AG, Vaduz, Liechtenstein; extra fine, 4–8 μm grit size, Kenda Unicus, Kenda AG, Vaduz, Liechtenstein). The fabricated ceramic blocks were intended to represent inlays with thicknesses of 2.0 mm, 2.5 mm, 3.0 mm, and 3.5 mm. The final dimensions of each ceramic block were determined using a digital caliper with an accuracy of 0.001 mm (Mitutoyo, Tokyo, Japan). To provide multiple measurements, the ceramic specimens were not acid-etched, silanated, or coated with an adhesive.

The ceramic blocks were cemented with light-cured adhesive cement, dual-cured adhesive cement mixed with an Automix syringe, and a preheated restorative RBC. A single-dose capsule of the latter RBC was preheated to 55 °C in an RBC warming device (Ena Heat Composite Heating Conditioner, Micerium, Avegno, Italy) for 15 min. Each capsule was heated once for the cementation of only one ceramic specimen. The resulting RBC temperature was measured using a non-contact infrared digital thermometer (TESTO 845, Testo Magyarország Kft., Budapest, Hungary). The infrared thermometer registered temperatures in an area as small as 1 mm^2^ (optical resolution of 75:1), with a resolution of 0.1/1 °C. The data sampling frequency was 10 measurements/s. The ceramic blocks were pre-warmed in a composite warming device to reduce heat dissipation during cementation.

During each cementation a light-emitting diode (LED) light curing unit (LCU) (LED.D, Woodpecker, Guilin, China; Λ = 420–480 nm; 8 mm exit diameter fiberglass light guide) was used in the standard mode for 40 s of exposure time. The LCU was powered by a line cord at room temperature (24 °C ± 1 °C). The position of the light guide tip was standardized to ensure that each sample received the same light beam character. A radiometer checkMARC radiometer (Bluelight Analytics, Halifax, NS, Canada) was used to monitor the radiant exitance (mW/cm^2^) of the LCU before and after exposure. The tip of the LCU was placed at a standard distance of 1 mm from the radiometer sensor.

### 4.2. Sample Preparation for Pulpal Temperature Measurements

**Figure 7:**
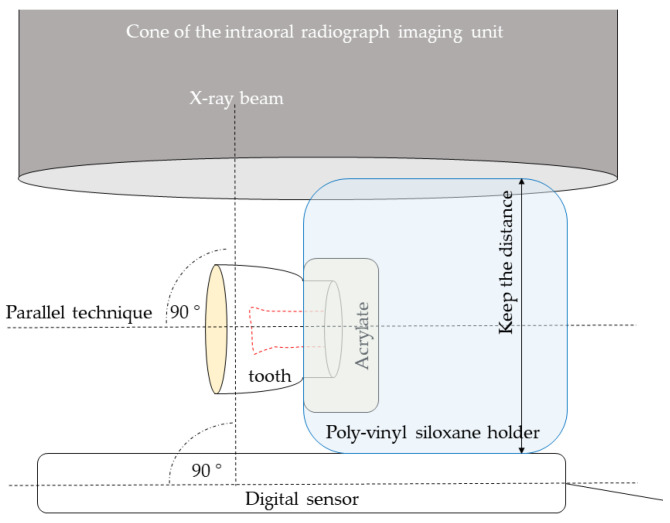
Schematic of the experimental set-up for radiograph taking during dentin thickness reduction.

A caries-free, freshly extracted, human mandibular third molar for use in this study was cleaned and kept in physiological saline at room temperature. All thermal measurements were performed on a single-tooth model to limit any effects of structural differences in the dental hard tissues [25]. The apices of the roots were cut 5 mm from the furcation to expose the root canals, and all the pulpal residues were removed with an endodontic file, which was followed first by irrigation with 5.25 weight% sodium hypochlorite solution (Chloraxid, Cerkamed, Stalowa Wola, Poland) and then saline (NaCl 0.9%, B. Braun, Melsungen, Germany), and was finally dried with paper points (DiaDent, Burnaby, BC, Canada). A hole was prepared on the mesial side of the tooth with a cylindrical diamond bur (836-012-FG cylinder diamond bur, 1.2 mm, medium; Meisinger USA, Centennial, CO, USA) to allow the insertion of the 0.5 mm diameter Cu/CuNi thermocouple probe (Type K thermocouple device; Ø = 0.5 mm; Cu/CuNi; TC Direct, Budapest, Hungary). The thermocouple sensor was positioned on the dentin at the top of the pulp chamber and assessed radiographically. To replicate the pulp tissue, the pulp chamber and root canal were injected with ECG gel (Aqua Sound Basic, Ultra-gel Hungary 2000, Budapest, Hungary). A flowable RBC (Filtek Supreme Flowable, 3M, St. Paul, MN, USA) was used to close the mesial hole and apical orifice, and the tooth was embedded in clear acrylic 1.0 mm below the cemento-enamel junction. The occlusal surface was prepared and polished flat, leaving dentin with a 2.5 mm thickness from the top of the pulp chamber. The occlusal thickness to be removed was estimated and controlled using digital intraoral radiography. To provide standard conditions during radiographic control of dentin reduction, the acrylic holder of the tooth was inserted in a poly-vinyl siloxane (Aquasil Ultra Plus, Dentsply Sirona, Charlotte, NC, USA) holder which was able to maintain the standard distance between the digital sensor and tube and provided correct position of the tooth for the parallel radiographic positioning technique (Figure 7).

After implementation of the first series of measurements with the different thicknesses of ceramic specimen and luting agent combinations, the dentin was reduced by 0.5 mm, resulting in a 2.0 mm dentin thickness. Following the conduction of the second series of measurements, the dentin thickness was further reduced by 0.5 mm, leaving behind 1.5 mm of dentin above the pulp chamber. For the last series of temperature registration, the dentin thickness was 1.0 mm thick after further reduction of 0.5 mm. The thickness of the remaining dentin was radiographically assessed. A cylindrical polytetrafluoroethylene (PTFE) mold with a crown diameter of ~12 mm and thicknesses of the four ceramic plates, with an inner hole of 6 × 6 mm, was fabricated to represent the axial walls of the cavity. The sample tooth was isolated with a rubber dam (Rubber Dam, Cerkamed, Stalowa Wola, Poland) supported by a frame and immersed in a water bath at 36.0 ± 0.5 °C. Temperature measurements were recorded using a digital thermometer (El-EnviroPad-TC, Lascar Electronics Ltd., Salisbury, UK) attached to the above-described thermocouple, with a resolution of 0.1 °C and a frequency of one measurement per second.

**Figure 8:**
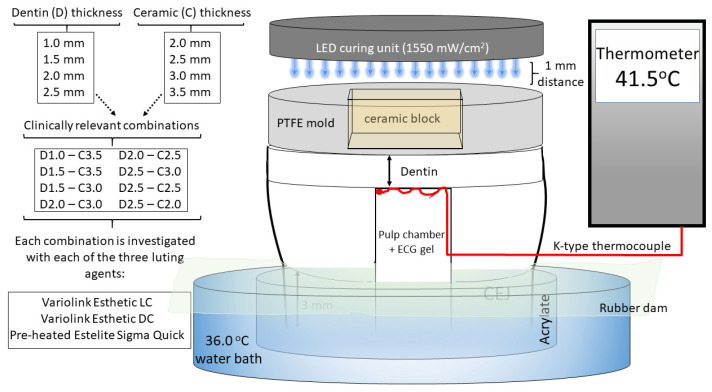
Schematic of the experimental set-up for pulpal temperature measurements.

First, the intrapulpal temperature changes during light exposure (40-s exposure) of dentin adhesive through the different dentin thicknesses using the empty molds were measured. Prior to dentin adhesive (Adper Single Bond 2, 3M ESPE, St. Paul, MN, USA; without acid conditioning) application, an agar/alcohol solution (3 wt% agar dissolved in 1:1 alcohol/water) was applied on the prepared dentin surface, allowing the polymerized adhesive/luting agent to be removed without remnants. Thereafter, the thermal effect of the LCU through the eight combinations of different thicknesses of dentin and ceramic was recorded using the digital thermometer. Temperature changes during cementation with the three resin-based luting agents were also measured (Figure 8).

A standard volume of the luting agent was applied to the center of the non-glazed ceramic surface, which was then centrally oriented to the tooth surface and manually (using the Optrasculpt instrument; Ivoclar Vivadent, Schaan, Liechtenstein) loaded with a 5-N load when resin adhesive cements were used and a 10-N load when the preheated restorative RBC was applied. Preliminary test results showed that manual placement of the ceramic specimen with a load of 5 or 10 N, depending on the type of luting agent, achieved a consistent layer thickness of 100 ± 10 μm. The load was measured using an algometer (Force Dial FDK 16, Wagner, Greenwich, USA). A microbrush was used to remove excess luting agent, which was photoactivated through the ceramic block for 40 s. After the cemented specimen was removed, its thickness was measured using a digital caliper. The luting agent film thickness was calculated from the difference between the thicknesses of the cemented specimen and the ceramic block alone. There were 24 groups of the various dentin/ceramic/luting agent combinations, and the temperature measurements were recorded five times for each group (n = 120).

As separating solution was used on the tooth surface and no ceramic surface treatment was applied, the polymerized luting agent could be removed from both the tooth and the ceramic block following the measurements, without leaving any deposits on either surface.

### 4.3. Differential Scanning Calorimetric (DSC) Measurements

A new series of the above-described ceramic blocks with thicknesses of 2.0 mm, 2.5 mm, 3.0 mm, and 3.5 mm were used for microcalorimetric measurements. Dentin slices were obtained from three third molars of a patient who was 19 years old. The teeth were sound, with no cracks or caries. The cleaned teeth were stored in saline at room temperature before testing, which took place within two days after extraction. To obtain dentin slices, the occlusal surface of the crown was horizontally removed using an orthodontic model trimmer (Gamberini, Bologna, Italy) under a water coolant. A minimum of 4 mm of dentin remained without exposing the pulp chamber. Dentin specimens with thicknesses of 1.0 mm, 1.5 mm, 2.0 mm, and 2.5 mm were sectioned using a precision cutter (IsoMet Low Speed Precision Cutter, Buehler, Lake Bluff, IL, USA) under a water coolant perpendicular to the long axis of the tooth. The enamel was removed to ensure that all sections contained only dentin, with final dimensions of 6 × 6 mm. The final dimensions of each dentin slice were measured using a digital caliper. Both ceramic and dentin specimens were weighed (ML-T Precision Balances, Mettler-Toledo, Greifensee, Switzerland).

Microcalorimetric measurements were carried out using a MicroSC microcalorimeter (Setaram Instrumentation, Caluire, France) in the differential scanning mode. Data were evaluated using the Calisto thermal analysis software (Setaram Instrumentation, Caluire, France). During the measurements, the samples were first equilibrated thermally for 5 min at 20 °C; then, the temperature was switched to 40 °C. The heat flow was recorded and plotted against time within 0–10 min. Considering that the sample holder itself needed 25 s to reach 40 °C after equilibration at 20 °C, data collected after 2 min were used for evaluation. Data evaluation was based on the following assumption: convective heat transfer between the sample holder of the microcalorimeter equipment and the samples occurred through the surface of the samples. The sample with higher heat conductivity requires less time to reach the thermal equilibrium at 40 °C. Accordingly, the data collected within the time range of 2–10 min were applied to estimate the heat conductivity, whereas the data collected within the time range of 0–10 min were applied to determine the heat capacities. The rate of the development of the thermal equilibrium at 40 °C was applied to compare the heat conductivities of the ceramic and dentin samples. The formation of thermal equilibrium was described by fitting the following equation (Equation (1)) to the measured data:

(1) $$heat\;flow=A\times e^{\frac{-t}{\tau }}$$

where *A* is the pre-exponential factor (proportional to the heat capacity); *t* is the time required; *τ* is the time constant describing the rate of formation of thermal equilibrium; and *e* is the base of the natural logarithm. Measurements were performed in triplicate for each sample.

### 4.4. Statistical Analysis

An earlier pilot study and a sample size formula were used to estimate the sample size [66].

The sample size formula is as follows: $n=\frac{{\left(z_{1-\frac{\alpha }{2}}+z_{1-\beta }\right)}^{2}{\left(s_{1}+s_{2}\right)}^{2}}{{\left(M_{1}+M_{2}\right)}^{2}}$ where z = standard score; α = probability of Type I error = 0.05; z_1−α/2_ = 1.96 for 95% confidence; β = probability of Type II error = 0.20; 1 − β = the power of the test = 0.80; z_1−β_ = value of standard normal variate corresponding to 0.80 value of power = 0.84; s_1_ = standard deviation of the outcome variable of group 1 = 0.64; s_2_ = standard deviation of the outcome variable of group 2 = 0.36; M_1_ = mean of the outcome variable of group 1; M_2_ = mean of the outcome variable of group 2; and (M_1_ − M_2_) = 0.5, if it is expected to detect a 0.5 °C difference between the two investigated groups as significant. Using the formula $N=\frac{2n}{1-0.1}$ the predicted sample size (n) was found to be 4.7 samples per group. According to the calculations, n = 5 per group sample size was indicated.

The SPSS v. 26.0 (SPSS, Chicago, IL, USA) software was used to perform statistical analyses. The normality of the data distribution was tested using the Kolmogorov–Smirnov test, followed by the application of parametric statistical tests. The differences in temperature changes were compared using one-way analysis of variance (ANOVA). Tukey’s post hoc adjustment was used for multiple comparisons for all the ANOVA models. To evaluate and explain the relative effect size on dentin and ceramic thicknesses, as well as the luting material as the independent variable, a general linear model (multivariate analysis) and partial eta-squared statistics were applied. Linear regression was used to determine the correlation between the dependent (temperature) and independent (dentin thickness, ceramic thickness, and luting material) variables. The statistical significance was set at *p* < 0.05.

## 5. Conclusions

The intrapulpal temperature rise may exceed the critical 5.5 °C threshold during ceramic inlay cementation, regardless of the dentin and ceramic thicknesses and the type of resin-based adhesive luting material used. The temperature values were predominantly influenced by the remaining dentin thickness, followed by the applied resin-based adhesive luting materials, and were least influenced by the ceramic thickness. The thermal conductivity of the dentin was 24% less compared to that of the ceramic, while the thermal capacity was 86% higher.

In order to provide safe dental treatment, a more conservative approach with more dentin preservation should be followed when it comes to the thermal effect applied to teeth during ceramic inlay cementations.

## Figures and Tables

**Table 1:** Materials, manufacturers, classification, and composition of the investigated adhesive resin cements and preheated resin-based composites.

Material (Code)	Shade	Manufacturer	Classification	Resin System	Filler	Filler Loading
Variolink Esthetic LC (VE_LC)	Light	Ivoclar Vivadent, Schaan, Liechtenstein	Light-curing adhesive resin cement	UDMA; 1,10-DDMA	0.04–0.2 μm ytterbium trifluoride and spheroid mixed oxide	38 vol% 64 wt%
Variolink Esthetic DC (VE_DC)	Light	Ivoclar Vivadent, Schaan, Liechtenstein	Dual-curing adhesive resin cement	UDMA; 1,10-DDMA	0.04–0.2 μm ytterbium trifluoride and spheroid mixed oxide	38 vol% 64 wt%
Estelite Sigma Quick (EQ_55 °C)	A1 enamel	Tokuyama Dental, Tokyo, Japan	Conventional submicron RBC preheated to 55 °C	BisGMA, TEGDMA	0.1–0.3 μm monodispersing spherical silica–zirconia filler; prepolymerized filler of silica-zirconia and copolymer	71 vol% 82 wt%

Abbreviations: RBC: resin-based composite; BisGMA: bisphenol-A diglycidil ether dimethacrylate; UDMA: urethane dimethacrylate; 1,10-DDMA: 1,10-dodecane dimethacrylate; LC, light-cure; DC, dual-cure; vol%, volumetric %; wt%, weight %.

## Data Availability

The data that support the findings of this study are available from the corresponding author upon reasonable request.
